# Spanish Validation of the *Conflict Tactics Scale-Parent to Child*: Assessing Non-Violent Discipline, Coercive Discipline, and Physical Aggression

**DOI:** 10.3389/fpsyg.2025.1579200

**Published:** 2025-06-04

**Authors:** Danilo Dominguez, Carles Pérez-Testor, Paula Benedico-Peydró, Aina Casarramona, Berta Aznar-Martínez

**Affiliations:** Faculty of Psychology, Education and Sports Sciences, Ramon Llull University, Barcelona, Spain

**Keywords:** child maltreatment, non-violent discipline, coercive discipline, physical aggression, psychological aggression, corporal punishment, physical maltreatment

## Abstract

**Introduction:**

There is a gap in the available Spanish-language instruments that specifically measure the behaviors or tactics used by parents in conflict or hostile situations with their children. The main objective of this study was to translate, adapt, and validate the Conflict Tactics Scale: Parent to Child (CTSPC) within the Spanish context, evaluating its psychometric properties, including exploratory factor analysis, confirmatory factor analysis, and internal consistency.

**Method:**

A sample of 700 parents (350 women and 350 men) aged between 18 and 69 years was used.

**Results:**

The Spanish version of the CTSPC consists of 21 items distributed across 3 dimensions: Non-Violent Discipline (*α* = 0.71), Coercive Discipline (α = 0.81), and Physical Aggression (α = 0.93). The findings indicated that 14.14% of participants reported having used physically aggressive behaviors toward their children at least once in their lifetime.

**Discussion:**

This instrument emerges as a valuable tool for identifying inappropriate tactics and behaviors employed by parents toward their children, contributing to the prevention of child maltreatment and raising parental awareness about how to educate children without resorting to violence.

## Introduction

Violence directed at children and adolescents constitutes a serious social, public health, and human rights issue, with a high global prevalence, affecting over one billion minors (World Health Organization, 2020; UNICEF, 2025). Child maltreatment occurs within the immediate environment of minors, particularly at the hands of parents and/or caregivers. It encompasses various forms of psychological, physical, and sexual abuse, as well as emotional and physical neglect, directed at individuals under the age of 18. These forms of maltreatment can cause both immediate and long-term harm to their health and development (World Health Organization, 2024; UNICEF, 2025).

Currently, three out of four children under the age of five are routinely disciplined through psychological abuse or corporal punishment by their parents and/or caregivers (World Health Organization, 2024; UNICEF, 2025). A World Health Organization (2022) global report estimated that 36% of children had experienced psychological abuse, 23% physical abuse, 16% of children had experienced neglect, and 18% of girls and 8% of boys sexual abuse (Save the Children, 2021; UNICEF, 2025). One global study measured the prevalence for sexual violence against children at super-region levels, estimating that approximately 12% of children had suffered from it in Southeast, Central and East Asia, Oceania, and Central and Eastern Europe, 26% of females in South Asia and 18% of males in Sub-saharan Africa (Cagney et al., 2025). Looking at other forms of violence against children in Europe, abuse affects 30% of children in terms of psychological abuse, 10% in the form of physical abuse and 20% in terms of physical neglect. According to a study made in the UK, approximately 11% of children experienced physical abuse, 12% emotional abuse and 15% neglect (Nation et al., 2023). Specifically in Spain, it is estimated that approximately 25% of adolescents have experienced child maltreatment in the past year by their parents and/or caregivers (Save the Children, 2021; UNICEF, 2025).

According to UNICEF (2025), the official data presented by the Unified Child Maltreatment Suspicions Registry (RUMI) reported 29,770 notifications of suspected child maltreatment, with emotional abuse (13,724 cases), physical abuse (9,044 cases), sexual abuse (5,449 cases), and neglect (19,979 cases). However, it is important to note that precise data on the number of children experiencing abuse or other forms of victimization by caregivers, such as corporal punishment, is not available, as many cases may not be properly communicated, detected, or reported. In a study conducted in the Portuguese context, fathers engaged in psychological aggression and corporal punishment more than mothers, while mothers were more likely to use severe and very severe physical assault (Abrahamyan et al., 2024). Additionally, current society still lacks full awareness that certain parenting methods involving shouting, insults, and physical punishment constitute forms of violence with serious physical and emotional consequences for children, who often normalize this violence and do not recognize themselves as victims. Therefore, it is necessary to provide parents with tools to educate children without resorting to violence (Save the Children, 2018; Save the Children, 2021; UNICEF, 2025).

In this context, it is essential to have instruments that assess the tactics or behaviors used by parents towards their children in conflict or hostile situations, in order to establish effective measures for prevention, detection, and intervention, thus preventing escalation into cases of violence. There are few instruments that address aspects related to the parent–child relationship, parenting styles, or family violence. These include the Escala de Normas y Exigencias (Fuentes et al., 1999), which assesses whether the parenting style is inductive, rigid, or punitive; the Escala de Apego (Fuentes et al., 1999), which measures the type of communication between parents and children; the Cuestionario de Funcionamiento Familiar (Ortega et al., 1999), which evaluates seven key factors of family dynamics: cohesion, harmony, communication, permeability, affectivity, roles, and adaptability; the Instrumento Balora (Arruabarrena Madariaga and Hurtado Pedroso, 2018), which assesses the risk of neglect or abandonment situations; and the Escala de Estilos de Socialización Parental en la Adolescencia (Musitu and García, 2004), which measures the dimensions of Acceptance-Involvement and Coercion-Imposition. However, these instruments do not allow for the specific identification of the behaviors adopted by parents in conflict situations.

The *Conflict Tactics Scale: Parent to Child* (CTSPC) (Straus et al., 1998) is a modified version of the Conflict Tactics Scale (CTS), originally developed by the same authors to assess behaviors and tactics used to resolve conflicts within intimate partner relationships (Straus, 1979). While the CTS has led to several adaptations focused on partner violence, such as the CTS-2 (Loinaz et al., 2012) and the M-CTS (Muñoz-Rivas et al., 2007), these versions are specifically designed to study the dynamics between partners in intimate or marital relationships and are not directly related to the assessment of parental behavior.

The CTSPC was developed to measure parents’ tactics or behaviors when facing conflicts or expressing hostility towards their children. The sample of the original study (Straus et al., 1998) consisted of 1,000 U. S. parents (66% women and 34% men) with an average age of 36.8 years, all having at least one child under the age of 18. The CTSPC (Straus et al., 1998) identifies three dimensions: Firstly, Non-Violent Discipline (NVD) evaluates the use of four disciplinary practices widely used as alternatives to corporal punishment (explanation, time-out, privilege removal, and substitutional activity). Secondly, the Psychological Aggression (PSY) scale measures verbal and symbolic acts carried out by the parent with the intention of causing psychological pain or fear in the child. Thirdly, the physical aggression (PAG) scale, which is subsequently divided into three subscales according to the severity of the behaviors: corporal punishment, physical abuse, and severe physical abuse. The corporal punishment subscale, which is meant to assess the low-severity aggression, can be used to calculate acts of minor physical assault for which parents are exempt from prosecution for assault, and refers to acts that have traditionally been expected as responses from parents to persistent misbehavior (Straus, 1994; Straus and Mathur, 1996). The physical abuse and severe physical abuse subscale assess high-severity behaviors, such as hitting or kicking a child (Straus et al., 1998).

This instrument has been previously validated in other languages, such as Portuguese in the Brazilian context (Reichenheim and Moraes, 2003) and Chinese in a sample from Hong Kong (Chan, 2012). As expected, each culture has its own particularities that may influence the adaptation of the scale. Therefore, when translated into other languages or used in different contexts, the scale has undergone modifications tailored to local characteristics. For example, the original instrument (Straus et al., 1998) proposes three scales, with the last one, the Physical Aggression Scale, divided into three subscales assessing severity (corporal punishment, severe abuse, and very severe abuse). However, the Portuguese version (Reichenheim and Moraes, 2003) suggests merging the severe and very severe abuse subscales into one, resulting in a total of four scales (non-violent discipline, psychological aggression, corporal punishment, and physical abuse) instead of three. Similarly, Cotter et al. (2018) validated the CTSPC in a sample of 110 parent–child dyads with a history of physical abuse and identified a four-factor model: Non-Violent Discipline, Psychological Aggression, Corporal Punishment/Minor Physical Assault, and Severe Physical Assault, instead of the original three scales. They also eliminated one item, reducing the total to 21 items. These discrepancies can be attributed to social and cultural differences, as the acceptance of certain educational methods, such as corporal punishment, varies according to the cultural norms and values of each country or society (Ranghetti and Milani, 2021).

The CTSPC has also been validated in Spanish within the Uruguayan context (de los Campos et al., 2008). In this adaptation, the authors removed two items from the original instrument, reducing the total to 20, and proposed a two-factor structure: the first, related to non-violent parenting practices, which includes some behaviors of moderate psychological and physical violence, and the second, linked to practices of more extreme violence. In this context, terms typical of Spanish as spoken in Uruguay, such as *en la cola*, *el piso*, or *cachetada*, were identified, whose meanings may differ from those in other Spanish varieties, such as that of Spain. This example highlights the importance of considering linguistic and cultural particularities in the transcultural validation processes of instruments, as an appropriate adaptation and validation require attention to the differences inherent in the various Spanish-speaking contexts in linguistic and cultural terms (Ariño Bizarro and Bernad Castro, 2022; Sobrino Triana, 2018).

As highlighted, this instrument has garnered the attention of a wide community of researchers globally, leading to its validation in multiple languages, including Spanish, though not specifically the Spanish variant of Spain and its own culture. In this context, the present study aims to contribute to the validation of this instrument in multiple languages, with a particular focus on Spanish from Spain, offering significant potential for facilitating cross-cultural comparisons and providing updated data from a Spanish-speaking sample in Spain. Moreover, it aims to address the existing gap in available Spanish-language instruments, as these do not allow for the specific evaluation of the behaviors or tactics employed by parents in conflict or hostile situations with their children. Consequently, the main objective of this study is to translate, adapt, and validate the *Conflict Tactics Scale: Parent to Child* (CTSPC) into Spanish (Spain), while conducting both exploratory and confirmatory factor analyses, as well as evaluating the instrument’s internal consistency. Additionally, the study seeks to provide pertinent data regarding the behaviors parents employ in conflict situations with their children in Spain.

## Method

### Participants

The present study was conducted in Spain and involved a total sample of 700 participants, aged between 18 and 69 years (*M* = 41.23; *SD* = 8.30). The sample was evenly distributed among 350 (50%) females and 350 (50%) males. Regarding the remaining sociodemographic characteristics, the sample exhibited a diverse composition, although certain profiles were more prominently represented. The majority of participants were married (60.3%), held a university degree (29.9%), and were employed at the time of the study (77.7%). In terms of income level, the most represented group reported medium-range earnings (31%), suggesting a tendency toward a middle-income socioeconomic status. Additionally, there was a predominance of individuals identifying as Caucasian (79.9%) and Christian (55.7%). Participants were parents with at least one child, whose ages ranged from 1 to 18 years (*M* = 9.85; *SD* = 4.82). For more detailed information about the sample, refer to Table 1.

**Table 1:** Sociodemographic data.

	Men		Women		Total	
	*n*	%	*n*	%	*n*	%
Marital status						
Married	237	(56,2%)	185	(43,8%)	422	(60,3%)
Coupled	79	(48,2%)	85	(51,8%)	164	(23,4%)
Single	15	(25%)	45	(75%)	60	(8,6%)
Divorced	16	(33,3%)	32	(66,7%)	48	(6,9%)
Widowed	3	(50%)	3	(50%)	6	(0,9%)
Educational level						
Elementary	2	(50%)	2	(50%)	4	(0,6%)
High school (12–16 yrs)	54	(50,5%)	53	(49,5%)	107	(15,3%)
High school (16–18 yrs)	55	(56,1%)	43	(43,9%)	98	(14%)
Inter. vocational training	42	(46,2%)	49	(53,8%)	91	(13%)
Adv. vocational training	53	(42,1%)	73	(57,9%)	126	(18%)
University studies	97	(48%)	105	(52%)	202	(29,9%)
Master	40	(62,5%)	24	(37,5%)	64	(9,1%)
PhD	7	(87,5%)	1	(12,5%)	8	(1,1%)
Employment status						
Employed	287	(52,9%)	256	(47,1%)	543	(77,6%)
Unemployed	23	(41,8%)	32	(58,2%)	55	(7,9%)
Self-employed	22	(51,2%)	21	(48,8%)	43	(6,1%)
Homemaker	2	(8,7%)	21	(91,3%)	23	(3,3%)
Retired	2	(28,6%)	5	(71,4%)	16	(2,3%)
Unable to work	6	(46,2%)	7	(53,8%)	13	(1,9%)
Student	4	(57,1%)	3	(42,9%)	7	(1%)
Annual salary						
No income	13	(29,6%)	31	(70,4%)	44	(6,3%)
Under 12.450 euros	20	(22,2%)	70	(77,8%)	90	(12,9%)
12.451–20.200 euros	57	(38%)	93	(62%)	150	(21,4%)
20.201–35.200 euros	124	(57,1%)	93	(42,9%)	217	(31%)
35.201–60.000 euros	96	(67,1%)	47	(32,9%)	143	(20,4%)
60.001 euros or more	40	(71,4%)	16	(28,6%)	56	(8,1%)
Ethnic group						
Caucasian	284	(50,8%)	275	(49,2%)	559	(79,9%)
Latin American	45	(44,6%)	56	(55,4%)	101	(14,4%)
Middle Eastern	12	(42,9%)	16	(57,1%)	28	(4%)
Afrodescendant	9	(75%)	3	(25%)	12	(1,7%)
Religious affiliation						
Christianity	197	(50,5%)	193	(49,5%)	390	(55,7%)
Atheism	78	(43,6%)	101	(56,4%)	179	(25,6%)
Agnosticism	52	(54,2%)	44	(45,8%)	96	(13,7%)
Other affiliations	23	(65,7%)	12	(34,3%)	35	(4,9%)

### Instruments

#### Conflict tactics scale: parent to child (CTSPC)

The *Conflict Tactics Scale: Parent to Child (CTSPC)* (Straus et al., 1998) was developed to measure the tactics or behavior of parents when facing conflicts or expressing hostility towards their children. The instrument comprises 22 items categorized into three scales: non-violent discipline (*α* = 0.70), psychological aggression (α = 0.60), and physical assault (α = 0.55). The physical assault scale is subsequently subdivided into three subscales based on the severity of the behaviors, namely corporal punishment, physical maltreatment, and severe physical maltreatment. An eight-category Likert scale is used to respond to the items, with options ranging from 0 (This has never happened) to 6 (More than 20 times in the past year), referring to the past year, and a category 7 (Not in the past year, but it happened before), which covers the entire lifetime (Straus et al., 1998). Scores are obtained by summing the midpoints of the response categories selected by the participant. For categories 0, 1, and 2, the midpoints are the same as the response category numbers. For category 3 (3–5 times), the midpoint is 4; for category 4 (6–10 times), it is 8; for category 5 (11–20 times), it is 15; and for category 6 (More than 20 times in the past year), it is 25 (Straus et al., 1998). Response category 7 (“Not in the past year, but it happened before”) is used in two ways: (1) When the goal is to obtain scores that reflect only the past year, category 7 is not included, as it does not refer to that time frame; therefore, scores range from 0 to 6. (2) To assess the “lifetime prevalence” of physical aggression—i.e., whether aggression has occurred at any point in life. In this case, respondents who select any response from 1 to 7 receive a score of 1 (yes), as category 7 also captures experiences prior to the past year, while those who respond 0 receive a score of 0 (no). This approach results in a dichotomous response format, allowing for the calculation of the percentage of individuals who reported not having engaged in violence (0) and those who did (1–7) at some point in their lives (Straus et al., 1998).

#### *Ad hoc* sociodemographic questionnaire

The participants provided information about sex, age, marital status, educational level, employment status, annual net income, ethnic group, and religious affiliation.

### Procedure

Using the Brislin (1970) procedure, one of the most commonly used methods to date, the English version of Straus et al. (1998) *CTSPC* was translated into Spanish by a Spanish-speaking translator, an expert in the field of violence. Subsequently, a back-translation into English was conducted by two independent English-speaking translators. After this step, discrepancies were discussed to reach a consensus and ensure the equivalence of the two versions. Table 2 shows the Spanish version of the *CTSPC*. After the translation, the instrument was sent to a company specializing in data distribution, collection, and analysis, adopting a quantitative approach, specifically CAWI (Computer Aided Web Interviewing), applied to an online panel. The platform used for conducting online surveys was one of third-party-owned aggregated panels. To ensure the representativeness of the sample, a stratified random sampling was implemented based on gender quotas, with fixed quotas at 50% for males and 50% for females. The inclusion criteria specified that participants must be residents of Spain, be legal adults, parents, and have children under the age of 18.

**Table 2:** Scales and internal consistency of the Spanish version of the CTSPC.

Spanish version of the *conflict tactics scales: parent to child* (CTSPC)	
NVD (*α* = 0.71)	
1	Explicarle por qué hizo algo mal.
2	Darle «tiempo para pensar» (o enviarlo/la a su habitación).
5	Decirle que haga otra cosa en lugar de hacer lo que estaba mal.
6*	Gritarle.
17	Quitarle privilegios o castigarlo/la.
CD (α = 0.81)	
3**	Zarandearlo/la.
8**	Darle un azote en las nalgas con la mano.
10	Insultarlo/la o decirle palabrotas.
14	Amenazar con darle un azote en el trasero o pegarle, aunque sin hacerlo.
16**	Pegarle en la mano, el brazo o la pierna.
21	Llamarlo/la tonto/a, vago/a o algo similar.
22**	Abofetearle la cara, la cabeza o las orejas.
PAG (α = 0.93)	
4	Golpearlo/la en las nalgas con un cinturón, un cepillo de pelo, un palo o algún otro objeto duro.
7	Darle un puñetazo o una patada fuerte.
9	Agarrarlo/la por el cuello y estrangularlo/la.
11	Darle una paliza, es decir, golpearlo/la repetidamente con todas tus fuerzas.
13	Quemarlo/la o escaldarlo/la a propósito.
15	Golpearle en una parte del cuerpo que no sean las nalgas con un cinturón, un cepillo de pelo, un palo o algún otro objeto duro.
18	Pellizcarlo/la.
19	Amenazarlo/la con un cuchillo o pistola.
20	Tirarlo/la al suelo.
Total (α = 0.77)	

(*) Belonged to the original PSY scale; (**) Belonged to the original PA scale (CP subscale).

To verify eligibility, identification documents were requested. The platform is characterized by its rigorous quality control and data integrity. A minimum time for completing surveys is estimated, and surveys that do not meet this standard are eliminated. Additionally, control questions are included to verify respondents’ attention, and the consistency of answers is checked to detect any inconsistencies. Surveys with contradictions or incoherent responses are discarded. Clear instructions are provided throughout the questionnaire to ensure respondents understand what is being asked in each question. These measures, combined with proprietary fraud detection technologies, ensure a precise and reliable data collection process. Regarding ethical issues, this project obtained the approval of the Ethics Department of Faculty of Psychology, Education and Sport Science, Blanquerna, Ramon Llull University (IN: 2122016D).

### Data analysis

For the analysis, the statistical software SPSS (v.26) was used. Firstly, the items underwent an exploratory factor analysis (EFA) to identify a factor structure that appropriately fits the theory. Secondly, the factor structure identified in the first analysis was validated through a confirmatory factor analysis (CFA). According to Hu and Bentler (1999), the following cutoff points per index were recommended to assess the model fit of the proposed scale: CFI ≥ 0.90 and RMSEA ≤ 0.06. Additionally, the internal reliabilities of the factors and the total scale score were examined using Cronbach’s alpha index. Finally, a Pearson correlation was conducted among the three resulting dimensions to determine whether there was a positive, negative, or no correlation. Descriptive statistics (frequency, percentages, mean, and standard deviation) were calculated to identify the sociodemographic characteristics of the sample and the various tactics employed by parents towards their children. Regarding the handling of missing data, it should be noted that all surveys included in the analysis were fully completed, with no missing data, and met the established quality control criteria. Cases that did not meet these criteria were excluded and replaced with new surveys that fully complied with the established requirements.

## Results

In this case, the validity evidence was based on internal structure (Sireci and Benítez, 2023) and was conducted by investigating how the relationships between the test items and underlying dimensions supported the intended interpretation of scores. This was achieved through the use of exploratory factor analysis (EFA) and confirmatory factor analysis (CFA):

To identify the empirical factorial structure of the Spanish version of the CTSPC (Straus et al., 1998), an exploratory factor analysis was conducted on its 22 items. Within the principal component method, we applied a Varimax rotation. Prior to the analysis, we computed the Kaiser-Meyer-Olkin (KMO) measure of sampling adequacy, which yielded a value of 0.83, considered acceptable. Additionally, Bartlett’s test of sphericity (χ2 (231) = 12112.535; *p* < 0.001) was performed, which yielded statistical significance (*p* < 0.05), thus supporting the appropriateness of the factorial analysis.

By applying Kaiser’s rule, we identified three factors with eigenvalues exceeding 1 in our analysis. These factors collectively explain 60.4% of the data’s variability, surpassing the 50% threshold, thereby substantiating the questionnaire’s validity as an instrument for measuring three distinct dimensions. The first dimension comprises a total of 9 items and accounts for over 37% of the variance in the original data. The second dimension includes 7 items and explains more than 16%. The third dimension consists of 5 items and encompasses approximately 6% of the variance.

The distribution of items across the different factors exhibited some variations compared to the structure proposed by the authors of the original scale (Straus et al., 1998). In our adaptation, we added an item to the NVD scale that originally belonged to PSY. The PAG scale has remained unchanged, except for the items from the CP subscale, which have been merged with the original PSY scale. This new dimension resulting from the merger of the original PSY scale and the CP subscale has been named “Coercive Discipline (CD).”

As shown in Table 3, we present the rotated component matrix, which details the items that make up each dimension along with their respective factor loadings. It is important to note that only items with loadings equal to or greater than 0.4 were retained, and item 12, being the only one with a factor loading below this threshold, does not appear in any dimension.

**Table 3:** Rotated component matrix.

Item	Components		
	1 Physical aggression (PAG)	2 Coercive discipline (CD)	3 Non-violent discipline (NVD)
11	**0.91**	0.115	0.026
13	**0.896**	0.168	0.008
15	**0.852**	0.19	−0.041
19	**0.847**	0.101	−0.005
9	**0.844**	0.187	−0.007
18	**0.821**	0.218	0.076
20	**0.793**	0.174	−0.052
7	**0.775**	0.106	0.061
4	**0.612**	0.139	0.116
21	−0.043	**0.817**	0.011
10	0.228	**0.796**	0.012
22	0.291	**0.711**	−0.005
14	0.088	**0.662**	0.298
16	0.312	**0.645**	0.149
3	0.309	**0.504**	0.288
8	0.243	**0.415**	0.355
6	−0.012	0.485	**0.488**
2	0.089	0.127	**0.698**
17	−0.013	0.317	**0.691**
1	−0.104	0.077	**0.686**
5	0.013	−0.035	**0.68**
12	0.24	0.38	0.261

Extraction method: Principal component analysis. Rotation method: Varimax with Kaiser normalization. We only retained those correlations higher than 0.4. The factor loadings corresponding to the items that comprise each dimension are highlighted in bold.

**Figure 1:**
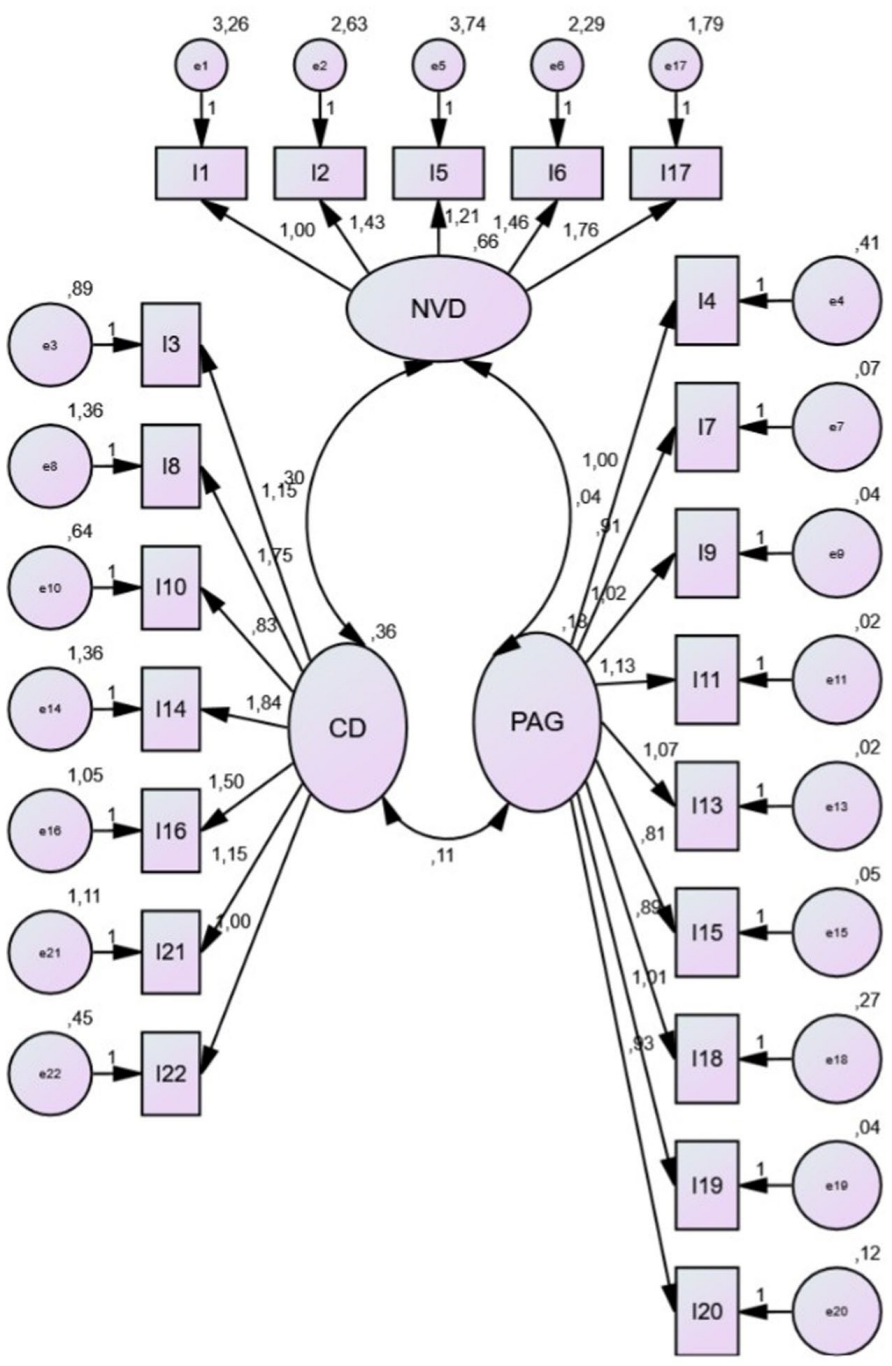
Confirmatory Factor Analysis on the CTSPC. The values represent standardized factor loadings and error variances.

A confirmatory factor analysis (CFA) (Figure 1) was conducted for the 21 items, with the model fit indices indicating a good fit (CFI = 0.910; RMSEA = 0.069), although there is room for improvement. Additionally, the internal consistency of the instrument was assessed using Cronbach’s alpha, which yielded a value of 0.77, indicating good overall reliability. An analysis of the internal consistency for each of the scales was also performed (see Table 2). Finally, the reliability of the scale was examined based on the removal or retention of each item, and it was found that all items contributed positively to the scale’s reliability.

Regarding the questionnaire results, as detailed in the Instruments section, there are two approaches to interpret response category 7 (“Not in the past year, but it happened before”). Following the first approach, in which category 7 is recoded as 0, the results show that, during the past year, non-violent discipline (NVD) was the most commonly used method, adopted by between 64.86 and 88.57% of participants (*M* = 27.06; *SD* = 24.20; Min = 0; Max = 125). Next, coercive discipline (CD) was reported by between 6.57 and 25.86% of participants (*M* = 4.74; *SD* = 11.91; Min = 0; Max = 148). Finally, physical aggression (PAG) was reported by only 1 to 6.43% of participants (*M* = 1.02; *SD* = 7.95; Min = 0; Max = 131). In the second approach, where category 7 is used to measure the “lifetime prevalence” of physical aggression, the results indicate that 14.14% of participants have used physically aggressive behaviors towards their children at least once in their lifetime.

Correlation analyses were conducted to examine the relationships among the three dimensions under study, assessing both convergent and discriminant validity (Campbell and Fiske, 1959; Elosua, 2003). The results revealed significant positive correlations between Non-Violence Discipline (NVD) and Coercive Discipline (CD) (*r* = 0.433; *p* < 0.001), supporting convergent validity, as both dimensions are linked to a common construct related to discipline. Additionally, a significant positive correlation was found between Coercive Discipline (CD) and Physical Aggression (PAG) (*r* = 0.421; p < 0.001), further supporting convergent validity, as both dimensions are associated with aggressive behaviors. However, no significant correlation was found between NVD and PAG (*r* = 0.061; *p* = 0.109), indicating discriminant validity. This result suggests that, although both dimensions are related to parental behavior, they are perceived as distinct constructs, as non-violent discipline and aggressive behaviors are considered separate aspects of parenting.

Finally, it is worth highlighting that significant sex-based differences were identified. Specifically, statistically significant differences emerged in the Non-Violent Discipline (NVD) subscale (*W* = 52504.000; *p* = 0.001), with women (*M* = 29.56; SD = 24.54) reporting a higher use of non-violent behaviors or tactics in conflict or hostile situations with their children compared to men (*M* = 24.55; SD = 23.63). Similarly, significant differences were found in the Physical Aggression (PAG) subscale (*W* = 65846.000; *p* = 0.004), with men (*M* = 1.79; SD = 11.12) reporting more frequent use of physically aggressive behaviors than women (*M* = 0.25; SD = 1.34). However, no significant sex differences were observed in the Coercive Discipline (CD) subscale (*p* > 0.05).

## Discussion

The main objective of this study was the translation, adaptation, and validation of the Conflict Tactics Scale: Parent to Child (CTSPC; Straus et al., 1998) into Spanish (Spain), with the aim of enhancing the availability of Spanish-language instruments to assess parental behaviors toward their children in conflict situations, given the lack of previously existing instruments of this nature. Additionally, it aimed to contribute to the validation of a multilingual instrument that facilitates cross-cultural comparisons and to promote international research in this field. Providing tools to assess the tactics used by parents is essential for the development of effective prevention, detection, and intervention strategies, thereby helping to prevent the escalation of conflict into violence. In Spain, there are few validated instruments that address the parent–child relationship, parenting styles, or family violence. Among the most notable are the Escala de Normas y Exigencias (Fuentes et al., 1999), Escala de Apego (Fuentes et al., 1999), Cuestionario de Funcionamiento Familiar (Ortega et al., 1999), Instrumento Balora (Arruabarrena Madariaga and Hurtado Pedroso, 2018), and Escala de Estilos de Socialización Parental en la Adolescencia (Musitu and García, 2004). However, none of these instruments specifically assess the behaviors adopted by parents in conflict situations.

Previous research (Cotter et al., 2018; de los Campos et al., 2008; Reichenheim and Moraes, 2003) has carried out the validation of CTSPC in other languages and contexts, adapting both the scales and items to local cultural characteristics, and making modifications such as the relocation of subscales and the removal of certain items. In all cases, the Corporal Punishment subscale was reclassified. In our adaptation, Corporal Punishment subscale has been merged with the Psychological Aggression scale, resulting in a new dimension: Coercive Discipline. This term reflects the interaction between psychological aggression and low-intensity physical maltreatment, which, while not reaching the levels of severe violence, can still have a significant negative impact on a child’s development. In this context, the findings of this study support the notion that psychological maltreatment, often minimized, can be equally or more harmful than physical maltreatment (Nguyen-Feng et al., 2024).

In addition, although the Psychological Aggression scale has incorporated items from the Corporal Punishment subscale, thus forming a new dimension, it has also eliminated or reallocated two other items. Firstly, item 12 (“Amenazar con mandarlo/a a vivir en otro lugar o con echarlo/a de casa”/“Said you would send him away or kick him out of the house”) was excluded due to its low factorial loading, as it did not show sufficient loading on any of the predefined dimensions during the exploratory factor analysis. Specifically, this item did not correlate significantly with any of the scales, which justifies its removal. Secondly, item 6 (“Gritarle”/“Shouted, yelled, or screamed at him”) was reallocated to the Non-Violent Discipline dimension, as the statistical analysis revealed that its factorial loading aligned more closely with this dimension than with Psychological Aggression. These decisions were based on the results obtained from the statistical analysis of the data. Such discrepancies can be attributed to variations in social and cultural norms, as the acceptance of certain educational methods may fluctuate according to the cultural values of each society (Ranghetti and Milani, 2021). In fact, in the validation carried out in Uruguay (de los Campos et al., 2008), the act of shouting was also considered a form of non-violent discipline. In this regard, it could be argued that, in Spanish-speaking populations, shouting is perceived as a quite common form of communication in conflict situations and is not necessarily socially conceptualized as an act of violence.

Finally, the Non-Violent Discipline and Physical Aggression scales have remained unchanged from their original versions (without subscales), except for the aforementioned adjustments in the Psychological Aggression scale, which have affected these other scales. Thus, the Spanish version of the CTSPC consists of a total of 21 items distributed across 3 dimensions: Non-Violent Discipline (NVD; *α* = 0.71), Coercive Discipline (CD; α = 0.81), and Physical Aggression (PAG; α = 0.93), significantly improving the reliability of the scales compared to the original version (Straus et al., 1998).

The majority of participants in this sample use non-violent discipline in conflict situations with their children. However, although coercive discipline and physical aggression are less frequent, in some cases they reach high values, suggesting that these strategies may be predominant in specific situations and applied in a more significant or intense manner. Moreover, the data indicate that non-violent discipline is more frequently used by mothers than by fathers, whereas physical aggression is reported more often by fathers. These findings underscore the importance of considering parental gender when examining disciplinary practices, as well as taking into account not only the frequency of specific behaviors but also the intensity with which they are implemented. It also highlights the need of sex-based interventions.

Furthermore, the correlation between the dimensions suggests that parents who use coercive discipline tend to combine aggressive and non-violent methods. However, those who adopt non-violent discipline show a clear tendency to avoid physical aggression, opting for approaches that prioritize calmness and peaceful conflict resolution. On the other hand, parents who resort to physical aggression tend to reject non-violent methods, favoring authoritarian and punitive approaches, which they perceive as more effective responses to conflict situations. At times, the distinction between Non-Violent Discipline (NVD) and Coercive Discipline (CD) may be unclear, underscoring the importance of promoting NVD as an educational approach, given the detrimental consequences that CD can have on child development. A similar phenomenon occurs between CD and Physical Aggression (PA), highlighting the need for the implementation of social and educational policies that raise awareness about the negative effects of PA on children’s well-being. Therefore, it is essential that such policies encourage educational practices that prevent the use of violence in parenting. Identifying these behaviors is the first step in breaking the normalization of violence in parenting, which would enable the provision of effective tools for parents to educate without resorting to violence (Save the Children, 2018; UNICEF, 2025).

The limitations of this study lie in the fact that, according to existing literature, child physical abuse is more common than generally perceived and reported (Save the Children, 2018; UNICEF, 2025). Consequently, certain high-violence behaviors, such as item 11 (“Darle una paliza, es decir, golpearlo/a repetidamente con todas tus fuerzas” / “Beat him up, that is you hit him over and over as hard as you could”), may be underrepresented, which could have led some participants to score such responses as 0. Further research is needed to explore the frequency and impact of severe physical abuse, as well as to refine measurement tools to better capture these behaviors.

In conclusion, despite the limitations of this study, the validation of the CTSPC in the Spanish context represents a crucial tool for identifying inappropriate tactics and behaviors of parents toward their children, enabling the implementation of prevention, detection, and intervention measures to prevent such behaviors from escalating into cases of physical or emotional violence. Given the lack of social awareness regarding parenting methods that involve violence, such as shouting, insults, or physical punishment, it is essential to provide parents with tools to educate without resorting to violence, fostering healthier and more respectful family environments. For future research, it would be pertinent to analyze the findings in relation to sociodemographic variables, such as educational level or employment status, to investigate potential differences in the use of disciplinary strategies. Although sample representativeness was addressed in this study, increasing the diversity of participants in subsequent research is necessary. Finally, it is recommended that these findings be further validated through additional studies conducted across diverse cultural and geographical contexts, both within Spain and internationally, in order to strengthen the instrument’s robustness and cross-cultural applicability.

## Data Availability

The original contributions presented in the study are included in the article/supplementary material, further inquiries can be directed to the corresponding author/s.
